# SMTP (*Stachybotrys microspora *triprenyl phenol) enhances clot clearance in a pulmonary embolism model in rats

**DOI:** 10.1186/1477-9560-10-2

**Published:** 2012-01-09

**Authors:** Weimin Hu, Ritsuko Narasaki, Naoko Nishimura, Keiji Hasumi

**Affiliations:** 1 Department of Applied Biological Science, Tokyo Noko University, 3-5-8 Saiwaicho, Fuchu, Tokyo 183-8509, Japan; 2 Research and Development Division, TMS Co., Ltd., 1-32-1-102 Fuchucho, Fuchu, Tokyo 183-0055, Japan

**Keywords:** plasminogen, fibrinolysis, thrombolysis, thromboembolism

## Abstract

**Background:**

*Stachybotrys microspora *triprenyl phenols (SMTPs) are a novel family of small molecules that enhance both activation and fibrin-binding of plasminogen. While their effects on fibrinolysis have been characterized *in vitro*, little is known about their activity *in vivo *with respect to plasminogen activation and blood clot clearance.

**Results:**

To select a potent SMTP congener for the evaluation of its action *in vitro *and *in vivo*, we tested several SMTP congeners with distinct structural properties for their effects on plasminogen activation. As a result, SMTP-7 (orniplabin) was found to have distinguished activity. Several lines of biochemical evidence supported the idea that SMTP-7 acted as a plasminogen modulator. SMTP-7 elevated plasma level of plasmin-α_2_-antiplasmin complex, an index of plasmin formation *in vivo*, 1.5-fold in mice after the intravenous injections at doses of 5 and 10 mg kg^-1^. In a rat pulmonary embolism model, SMTP-7 (5 mg kg^-1^) enhanced the rate of clot clearance ~3-fold in the absence of exogenous plasminogen activator. Clot clearance was enhanced further by 5 mg kg^-1 ^of SMTP-7 in combination with single-chain urokinase-type plasminogen activator.

**Conclusions:**

Our results show that SMTP-7 is a superior plasminogen modulator among the SMTP family compounds and suggest that the agent enhances plasmin generation *in vivo*, leading to clearance of thrombi in a model of pulmonary embolism.

## Background

The plasminogen/plasmin system plays a central role in blood clot lysis [1,2]. Plasminogen is a single-chain glycoprotein consisting of an N-terminal PAN domain, five homologous kringle domains, and a trypsin-like serine protease domain. Plasminogen is converted to the active enzyme plasmin by the specific cleavage of the Arg^561^-Val^562 ^bond by tissue-type plasminogen activator (t-PA) or urokinase-type plasminogen activator (u-PA). The binding of plasminogen to fibrin and cell surfaces, mediated by kringle domains in plasminogen, localizes fibrinolytic activity on fibrin and cell surfaces [3]. Plasminogen adopts a tight conformation due to an intramolecular interaction between a lysine residue (Lys^50 ^and/or Lys^62^) in the PAN domain and a lysine binding site in the fifth kringle domain [4,5]. The tight conformation of the plasminogen molecule attenuates its activation and interaction with fibrin and cellular receptors [3,6]. Lysine analogs such as 6-aminohexanoic acid bind to lysine binding sites in kringle domains and induce a large-scale conformational change in plasminogen [7,8], facilitating its activation by plasminogen activators. However, lysine analogs inhibit plasminogen binding to fibrin or cell surface receptors and, therefore, inhibit fibrinolysis. Fibrin is not only a substrate of plasmin but also a cofactor of plasminogen activation. Upon binding to fibrin, plasminogen undergoes conformational change to become susceptible to activation [9]. Further, partial degradation of fibrin by plasmin generates C-terminal lysines, resulting in the accumulation of more plasminogen to degrading fibrin to accelerate fibrinolysis.

Thus, conformational regulation of plasminogen is implicated in the localization and activation of plasminogen. This mechanism suggests that pharmacological modulation of plasminogen conformation will regulate local plasmin production [10]. We recently identified a series of small-molecule modulators of plasminogen activation. These compounds, which are structurally unrelated to lysine, enhance plasminogen activator-catalyzed plasminogen activation. Unlike lysine analogs, these modulators increase plasminogen-fibrin binding [11]. SMTPs (*Stachybotrys microspora *triprenyl phenols) and staplabin are the representatives of the "nonlysine-analog" plasminogen modulators. [11-20]. In this paper, we show that SMTP-7 (orniplabin), one of the most potent congeners, increases plasmin generation *in vivo *and promotes clot clearance in a rat pulmonary embolism model. These activities provide bases of the therapeutic activity of SMTP-7 toward thrombotic cerebral infarction [21-23].

## Results

### Activities of the SMTP congeners in vitro

**Figure 1:**
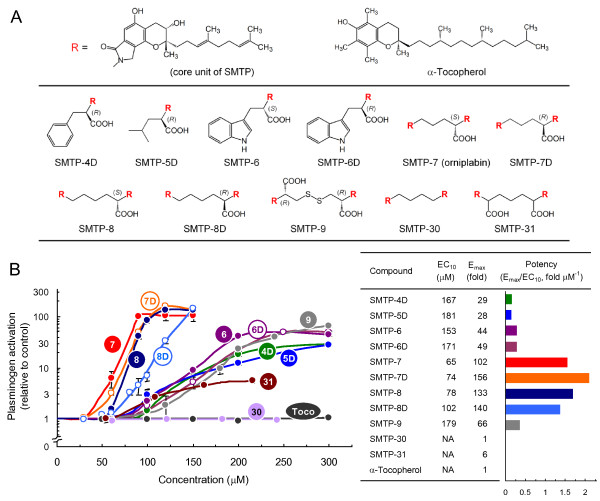
**Structure-activity relationships of SMTP congeners**. (A) Structures of SMTP congeners tested. The core unit of SMTP congeners is represented by R. (B) The activation of plasminogen was assayed in the presence of the indicated concentrations of SMTP congeners. Number in circle represents the SMTP number. *Toco*, α-tocopherol. Each value represents the mean ± SD from triplicate determinations. Relative to control values are shown. Summary of the results are shown in the right panel, where EC_10 _represents the concentration (μM) of SMTP that causes 10-fold enhancement of plasminogen activation and E_max _the maximum level of the enhancement (fold increase in plasminogen activation compared with control). E_max _and the reciprocal of EC_10 _are independent indexes that represent the potency of the compound. The ratio E_max_/EC_10 _represents comprehensive potency. *NA*, not available (due to that enhancement did not reach 10-fold at concentrations tested).

We assessed structure-activity relationships of SMTPs to select a congener to be studied in detail. SMTP congeners identified so far are roughly classified into two groups. One is the single-unit congener, consisting of the core triprenyl phenol unit and an *N*-linked side-chain, which is a side-chain of an amino acid (Figure 1A). The other group is the two-unit congener, consisting of two core units bridged by a diamine (Figure 1A). Among the 8 congeners tested, two-unit congeners (SMTP-7, -7D, -8, and -8D) were more active than single-unit congeners (SMTP-4D, -5D, -6, and -6D) in enhancing u-PA-catalyzed plasminogen activation (Figure 1B). α-Tocopherol, which has a structure resembling the core unit of SMTP (Figure 1A), was inactive (Figure 1B). To characterize further, 3 additional two-unit congeners, SMTP-9, -30, and -31 (Figure 1A), were synthesized (additional file 1). SMTP-9 and -31, which had two carboxyl groups, were less active than congeners with one carboxyl group (Figure 1B). SMTP-30, which had no carboxyl group, was essentially inactive (Figure 1B). Thus, the number of carboxyl group affects the activity of two-unit SMTP congeners. Based on these results, SMTP-7 was selected for detailed characterization.

### Characterization of the action of SMTP-7 in vitro

**Figure 2:**
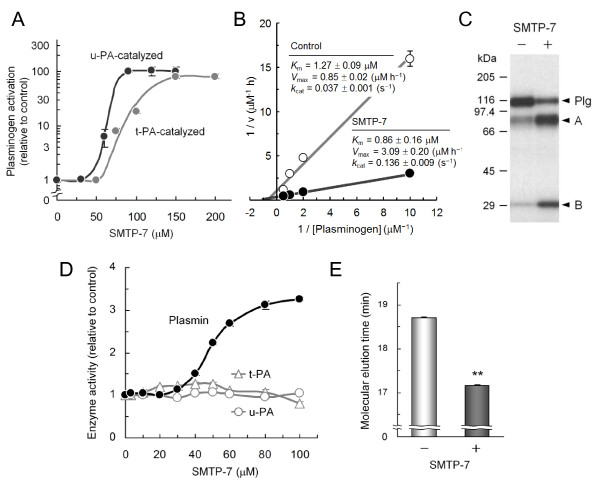
**Characterization of SMTP-7 *in vitro***. (A) Effects on plasminogen activation by t-PA and u-PA. Plasminogen activation was assayed using t-PA or u-PA in the presence of the indicated concentrations of SMTP-7. (B) Kinetic measurements of u-PA-catalyzed plasminogen activation in the presence of SMTP-7 (100 μM). Parameters shown were obtained from triplicate determinations. (C) Effect on plasminogen conversion to plasmin. u-PA-catalyzed ^125^I-plasminogen conversion to plasmin was assayed in the presence or absence of SMTP-7 (100 μM). Positions of plasminogen (*Plg*) and A- and B-chains of plasmin are shown. (D) Effects on amidolytic activities of plasmin, t-PA, and u-PA. Activity of each enzyme was determined using a fluorogenic substrate in the presence of the indicated concentrations of SMTP-7. (E) Size-exclusion chromatography. Alexa 488-labeled plasminogen was eluted in the absence or presence of SMTP-7 (120 μM). **, *P *< 0.05 by Student's *t*-test. Error bars in panels A, B, D, and E represent SD from triplicate determinations.

SMTP-7 enhanced t-PA-catalyzed plasminogen activation as well as the activation catalyzed by u-PA (Figure 2A). In the kinetic determinations of u-PA-catalyzed plasminogen activation, SMTP-7 markedly increased *V*_max _and slightly decreased *K_m_*, resulting in a large increase in *k*_cat _(Figure 2B). The increase in plasmin generation was confirmed by SDS-polyacrylamide gel electrophoresis (Figure 2C). Amidolytic activities of u-PA and t-PA were not affected by SMTP-7 (Figure 2D). While SMTP-7 moderately elevated the activity of plasmin (Figure 2D), the magnitude (~3-fold) was smaller than that of plasminogen activation (~100-fold). In size-exclusion chromatography, the molecular elution time of plasminogen was slightly shortened in the presence of SMTP-7 (Figure 2E), suggesting that an increase in apparent molecular volume was induced by SMTP-7. Taken together, these properties of SMTP-7 are consistent with the idea of a plasminogen modulator, which increases plasminogen activation by affecting the conformational status of plasminogen [10].

### SMTP-7 increases plasma Pm-AP level in mice

**Figure 3:**
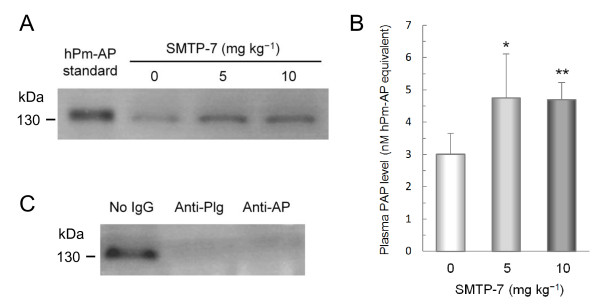
**Effect of SMTP-7 on plasma level of Pm-AP in normal mice**. Mice were intravenously injected with SMTP-7 at a bolus dose of 5 mg kg^-1 ^(*n *= 5) or 10 mg kg^-1 ^(*n *= 5). After 60 min, blood was drawn to determine Pm-AP levels in plasma by fibrinogen zymography. Control animals (*n *= 5) received saline. (A) Representative zymogram. Plasmas from 5 animals in each group were combined and aliquots (2 μl) of the mixtures were subjected to zymography. Human Pm-AP (*hPm-AP*, 1.7 ng) was resolved as the standard. (B) The summary of the quantification of Pm-AP. Plasma (2 μl) from each of the five mice was independently subjected to zymographic analysis. Data were calibrated by comparing the intensity of each lysis band with that of the standard human Pm-AP. The mean + SD of the data obtained from each animal is shown. *, *P *< 0.05 and **, *P *< 0.01 compared with control by the Dunnett's multiple comparison test. (C) Authenticity confirmation. The mixture of plasma in the 5 mg kg^-1 ^SMTP-7 group was treated with anti-plasminogen IgG (*Anti-Plg*), anti-α_2_-antiplasmin IgG (*Anti-AP*), or buffer before the zymographic determination of Pm-AP.

To assess plasminogen activation *in vivo*, we determined the level of Pm-AP as an index of plasmin generation. Pm-AP was determined by fibrinogen zymography, in which human and mouse Pm-AP appeared as lysis bands at ~140 and ~130 kDa, respectively (additional file 2). When SMTP-7 was administered to normal mice (5 and 10 mg kg^-1^, bolus intravenous injections), the level of Pm-AP in plasma was significantly increased (~1.5-fold, *P *< 0.05) (Figures 3A and 3B). Both anti-plasminogen IgG and anti-α_2_-antiplasmin IgG decreased the intensity of the lysis band, supporting that the lysis band represented Pm-AP (Figure 3C and additional file 2). These results suggest that SMTP-7 enhances plasmin generation *in vivo*.

### SMTP-7 enhances clot clearance in a rat pulmonary embolism model

**Figure 4:**
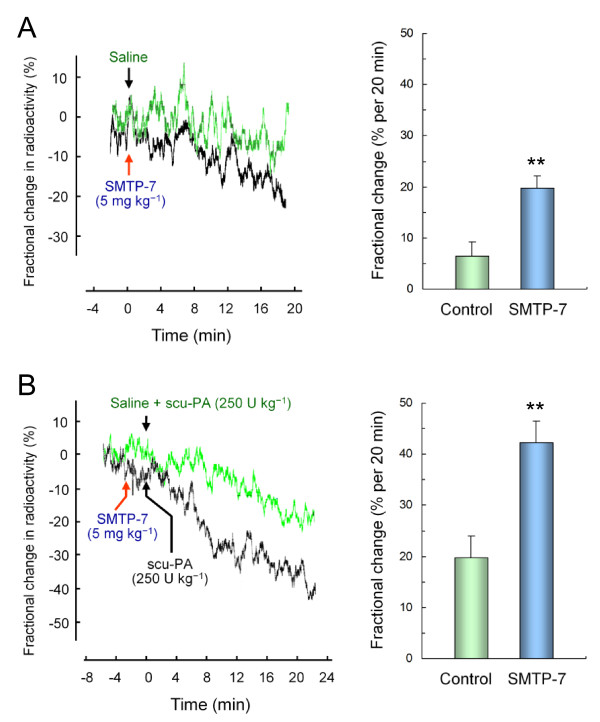
**Effect of SMTP-7 on clot clearance in a rat pulmonary embolism model**. Rats were injected intravenously with ^125^I-labeled plasma clots. The clearance of the clots was monitored before and after the treatments with SMTP-7 and/or scu-PA. (A) SMTP-7 (5 mg kg^-1^; *n *= 6) was administered intravenously ~20 min after the injection of ^125^I-labeled plasma clot. Control animals (*n *= 6) received saline. Radioactivity over the thorax was monitored (one of the 6 traces is shown in the left panel) and the fractional change in radioactivity was summarized (right panel). (B) SMTP-7 (5 mg kg^-1^), followed by 250 U kg^-1 ^of scu-PA (Thrombolyse^®^), was administered intravenously ~20 min after the clot injection (*n *= 6). One unit of Thrombolyse^® ^is comparable to ~60 IU of tcu-PA in fibrinolytic activity. In control animals (*n *= 6), scu-PA was given ~20 min after the clot injection. Arrow denotes time at which the administration was made. **, *P *< 0.01 compared with control by the Student's *t*-test with normally distributed variables.

SMTP-7 was evaluated further in a rat pulmonary embolism model, in which animals were injected with small particles of ^125^I-labeled plasma clots. The clots predominantly distributed over the lungs, and its clearance was monitored continually as the decay of radioactivity in the thorax. Based on the effects of SMTP-7 on the Pm-AP accumulation in mice (Figure 3), the dose of 5 mg kg^-1 ^was used. In control animals given saline alone, the clearance of ^125^I-plasma clots occurred slowly (6.4 ± 2.8% per 20 min) (Figure 4A). The treatment with SMTP-7 (5 mg kg^-1^, bolus intravenous injection) significantly increased the rate of clot clearance (19.8 ± 2.4% per 20 min) (Figure 4A). This increase was comparable to that (19.8 ± 4.1% per 20 min) brought by scu-PA (250 U kg^-1^, bolus intravenous injection) (Figure 4B). The combination of SMTP-7 (5 mg kg^-1^) and scu-PA (250 U kg^-1^) increased the clot clearance rate to 42.3 ± 4.2% per 20 min (Figure 4B).

### SMTP-7 does not increase bleeding in normal mice

Effects of SMTP-7 on bleeding and rebleeding were assessed by a tail amputation assay in normal mice. SMTP-7 was tested at the pharmacological dose (5 mg kg^-1^) and a higher dose (30 mg kg^-1^). Neither a statistically significant prolongation of bleeding time nor an increase in bleeding volume was observed at both doses (additional file 3).

## Discussion

In this study, we demonstrate that SMTP-7, one of the two-unit SMTP congeners, is a plasminogen modulator effective in plasminogen activation and clot clearance. Among the SMTP congeners tested, the two-unit congeners are more active than the single-unit congeners. Of the two-unit congeners tested, analogs with two carboxylic acid groups in the *N*-linked side-chain are weaker than those with one carboxylic acid group. The analog with no carboxylic acid in the *N*-linked side-chain is essentially inactive. Thus, the importance of both the core triprenyl phenol unit and a carboxylic acid group in the *N*-linked side-chain is demonstrated.

The action of SMTP-7 to enhance plasminogen activation conforms to the idea of zymogen modulators [10] based on the following observations: (i) SMTP-7 does not affect the enzymatic activity of u-PA and t-PA but increases plasminogen activation catalyzed by u-PA and t-PA; (ii) SMTP-7 alters conformational status of plasminogen, as evidenced by a change in molecular elution time on analytical size-exclusion chromatography. SMTP-7 enhances the conversion of plasminogen to the two-chain plasmin. This activity, along with the augmentation of the catalytic activity of plasmin (~3-fold), results in the apparent increase (up to 100-fold) in plasminogen activation assessed by using a chromogenic substrate. Kinetic data demonstrate that SMTP-7 increases *V*_max _of u-PA-catalyzed plasminogen activation with a slight decrease in *K_m_*, suggesting that faster turnover of the enzyme is allowed on the SMTP-7-modulated substrate compared with a native substrate.

The administration of SMTP-7 to normal mice resulted in an increase in the level of Pm-AP in plasma. Since plasmin generated *in vivo *is rapidly inactivated by α_2_-antiplasmin to afford Pm-AP, the level of Pm-AP represents the generation of plasmin *in vivo *[24]. SMTP-7 does not affect the rate of Pm-AP formation when plasmin is incubated with α_2_-antiplasmin in a purified system (unpublished observation). Thus, the finding that SMTP-7 increases plasma level of Pm-AP in mice suggests that SMTP-7 increases plasminogen activation *in vivo*. SMTP-7 is effective in promoting the clot clearance in the rat pulmonary embolism model. Compared with the spontaneous clot clearance rate, a 3-fold increase in the rate is brought by the administration of SMTP-7 at 5 mg kg^-1^, a dose that is effective in elevating plasma Pm-AP level. The plasma concentration of SMTP-7 at this dose just after the intravenous injection is expected to be ~100 μM. The rate of clot clearance after the SMTP-7 injection is comparable to the rate brought by the injection of scu-PA at 250 U kg^-1^. SMTP-7 synergistically enhances the clot clearance in combination with scu-PA. Thus, these properties of the SMTP-7 action *in vivo *conform to its activity *in vitro*.

One possible drawback of SMTP-7 may be systemic hyperfibrinolysis and following hemorrhage as that observed in disseminated intravascular coagulation. However, our preliminary studies suggest that these are unlikely to occur at a pharmacological dose (<10 mg/kg), since bleeding time and rebleeding volume in a mouse tail amputation assay were not changed statistically by the SMTP-7 administration at the pharmacological dose (5 mg kg^-1^) and a higher dose (30 mg kg^-1^) (additional file 3). This moderate effect of SMTP-7 on hemorrhage can be explained by that SMTP-7 is a zymogen modulator and its action depends on endogenous plasminogen activators, the availability of which is physiologically regulated.

Plasminogen activators such as recombinant t-PA are important drugs treating acute thrombotic stroke and myocardial infarction [25,26]. Recent investigations have demonstrated that SMTP-7 is quite effective in ameliorating thrombotic stroke in models of mouse and gerbil [21-23]. The data in this paper provide bases of the therapeutic activity of SMTP-7 in these models. It is of note that SMTP-7 is effective in the treatment after 3-6 h of the thrombotic stroke induction, whereas t-PA is ineffective when treated after 3 h [21,22]. The difference in the therapeutic efficacy between SMTP-7 and t-PA is partly explained as that SMTP-7 reduces inflammatory and oxidative responses associated with thrombotic ischemia [22,23,27]. t-PA is reported to induce cerebral inflammation and neuronal cell death by directly interacting with low density lipoprotein receptor-related protein and *N*-methyl-D-aspartate receptor [28,29]. In consistent with these observations, hemorrhagic transformation is reduced by SMTP-7, while it is increased by t-PA [22]. Thus, SMTP-7 can be a unique agent that aid in the treatment of thrombotic complications.

## Conclusion

Our results show that SMTP-7 is a superior plasminogen modulator among the SMTP family compounds and suggest that the agent enhances plasmin generation *in vivo*, leading to clearance of thrombi in a model of pulmonary embolism. These results provide mechanistic bases for the recent findings that SMTP-7 has a profound activity in treating thrombotic stroke in animal models.

## Methods

### Materials

Human native plasminogen was isolated by lysine-Sepharose affinity chromatography from frozen citrated plasma. The source of the following reagents were: single-chain u-PA (scu-PA; Thrombolyse^®^) from Mitsubishi Pharma (Osaka, Japan); high molecular weight u-PA (1.47 × 10^5 ^IU mg^-1^) from JCR Pharmaceuticals (Kobe, Japan); two-chain t-PA (7.0 × 10^5 ^IU mg^-1^) from Biopool (Umeå, Sweden); sheep anti-mouse plasminogen IgG from Haematologic Technologies (Essex Junction, VT, USA); rabbit anti-mouse α_2_-antiplasmin IgG from Merdian Life Science (Saco, ME, USA); human fibrinogen and human α-thrombin from Sigma (St. Louis, MO, USA); carrier-free Na^125^I from Amersham. Radioiodination of fibrinogen and plasminogen was performed using the iodine monochloride method. Upon trichloroacetic acid treatment, more than 95% of radioactivity in the fibrinogen and plasminogen preparations precipitated with protein. When treated with thrombin, approximately 70% of the radioactivity in ^125^I-fibrinogen was incorporated into the resulting clots.

### SMTP congeners

All the SMTP congeners used in this study were produced by *S. microspora *IFO 30018. SMTP-4D, -5D, -6, -6D, -7, -7D, -8, and -8D were isolated as described previously [15,17]. SMTP-9, -30, and -31 were originally isolated as described in additional file 1. In experiments *in vitro*, SMTPs were dissolved directly in buffer A (50 mM Tris-HCl, 100 mM NaCl, and 0.01% Tween 80, pH 7.4). In animal experiments, SMTP-7 was dissolved in saline by adjusting pH ~9 with dilute NaOH.

### Assay for plasminogen activation

Plasminogen activation was determined by measuring the initial velocity of plasmin generation using H-Val-Leu-Lys-*p*-nitroanilide (Bachem, Bubendorf, Switzerland), a chromogenic substrate for plasmin. A reaction mixture consisting of 50 nM plasminogen, 50 IU ml^-1 ^u-PA (or 200 IU ml^-1 ^t-PA) and 0.1 mM of the substrate in 50 μl of buffer A was incubated in a well of a 96-well microplate at 37°C for up to 40 min. Absorbance at 405 nm was measured with an interval of 1 to 2 min. From the slope of the plots of A_405 _nm versus *t*^2^, the initial velocity of plasmin generation was calculated. In the experiment to determine kinetic parameters, assays were performed with varying concentrations of plasminogen (0.5-2 μM) and a fixed concentration of u-PA (50 IU ml^-1^) in the presence or absence of SMTP-7 (100 μM). Since SMTP-7 at 100 μM enhanced plasmin activity toward H-Val-Leu-Lys-*p*-nitroanilide by 3.26-fold (*see *Figure 2D), this factor was taken into account in the process of the calculation of plasmin generation velocities.

Plasminogen activation was alternatively assayed by determining the conversion to the two-chain form. ^125^I-Plasminogen (100 nM) was incubated with u-PA (50 IU ml^-1^) and aprotinin (100 kallikrein inhibitor units ml^-1^) in buffer A at 37°C for 30 min. The mixture was resolved on SDS-polyacrylamide gel electrophoresis under reducing conditions. The gel was stained with Coomassie Brilliant Blue R-250.

### Assay for activities of plasmin, u-PA, and t-PA

Amidolytic activities of plasmin (10 nM), u-PA (1 IU ml^-1^), and t-PA (2000 IU ml^-1^) were determined at 37°C in buffer A using 10 μM of H-Val-Leu-Lys-7-amino-4-methylcoumarin, *t*-butyloxycarbonyl-Glu-Gly-Arg-7-amino-4-methylcoumarin, and succinyl-Phe-Ser-Arg-7-amino-4-methylcoumarin, respectively.

### Size-exclusion chromatography

Size-exclusion chromatography was performed using a TSK-Gel G-3000SW column (7.5 × 600 mm, TOSOH, Tokyo, Japan) equilibrated with buffer A in the presence or absence of SMTP-7 (120 μM). Plasminogen labeled with Alexa Fluor^® ^488 (Molecular Probes, Eugene, OR, USA) (10 μg) was resolved at room temperature at a rate of 1 ml/min. The elution was monitored using a fluorescence detector with an excitation at 495 nm and an emission at 520 nm.

### Animal Experiments

All of the animal protocols were approved by the institutional animal care committee of Tokyo Noko University. Male Wistar rats and male ICR mice were obtained from Japan SLC (Hamamatsu).

### Determination of plasmin-α_2_-antiplasmin complex (Pm-AP)

Male ICR mice (7 weeks of age) received intravenous SMTP-7 or saline. After 60 min, blood was collected from inferior vena cava in 13 mM sodium citrate. Plasma was rapidly prepared by centrifugation and mixed with one volume of buffer B (125 mM Tris-HCl, pH 6.8, 4% (w/v) SDS, 20% (w/v) glycerol, and 0.004% (w/v) bromophenol blue). In some experiments, plasma was pretreated with ant-plasminogen IgG (0.6 mg ml^-1^) or anti-α_2_-antiplasmin IgG (3 mg ml^-1^) for 30 min at room temperature before mixing with buffer B. The mixture (equivalent to 1-2 μl of plasma) was resolved on nonreduced SDS-polyacrylamide gel electrophoresis on a 7.5% gel containing fibrinogen (2 mg ml^-1^) at 4°C. As the standard for the calibration of Pm-AP in plasma samples, 1.7 ng of human Pm-AP (prepared by incubating 120 nM human plasmin and 600 nM human α_2_-antiplasmin for 30 min at 37°C in buffer A) was resolved on the same gel. After electrophoresis, the gel was cut at the position of ~90 kDa, and the upper half was washed with Triton X-100 (2.5 %, w/v) and incubated in 50 mM Tris-HCl, pH 8.3, and 100 mM glycine for 60 h at 37°C. (The lower half gave a strong lysis band at ~70 kDa, possibly due to plasma kallikrein) Gels were stained with Coomassie Brilliant Blue R-250. The lysis zones due to protease activities appeared as unstained bands on a blue background. Human Pm-AP gave a lysis band at ~140 kDa, while the mouse counterpart afforded a band at ~130 kDa (additional file 2). The scanned image of the stained gel was reversed for presentations. The band intensity was determined using Scion image. The amounts of Pm-AP in test samples were calibrated by comparing intensities of lysis bands of samples with that of the standard, and data were expressed as human Pm-AP equivalent (nM in plasma).

### Preparation of ^125^I-plasma clot particles

Platelet-poor plasma from male Wistar rats was mixed with ^125^I-fibrinogen (139 μg ml^-1^, ~2.5 MBq) and α-thrombin (1 IU ml^-1^) in the presence of 44 mM CaCl_2_. After incubation at 37°C for 120 min, the resulting plasma clot was washed thrice with saline and powdered in a mortar under liquid nitrogen, followed by homogenization 4 strokes in 2.7 ml saline using a Potter Elvehjem homogenizer with a Teflon pestle (13-mm in diameter). The suspension was left at room temperature for 30 min, and the resulting precipitates were homogenized again. This operation was repeated once more. The combined homogenates were settled for 30 min, and the resulting supernatant was centrifuged at 20 × g for 3 min to obtain pellet consisting of clot particles of 10-100 μm in diameter.

### Measurement of clot clearance in the lungs

Male Wistar rats weighing ~120 g were kept at 22°C with normal chaw for 1-7 days before the use in experiments. Rats were anesthetized with urethane and chloralose (750 and 65 mg kg^-1^, respectively, i.p.), and a probe (equipped with a 10-mm collimator) of a model TCS-163 NaI scintillation survey monitor (Aloka, Tokyo, Japan) was placed above the thorax. A suspension of ^125^I-plasma clot particles (75 μl kg^-1^; ~1.2 × 10^7 ^Bq per animal) was injected simultaneously with heparin (165 units kg^-1^) and NaI (3.3 mg kg^-1^) into a caudal vein. The ^125^I-plasma clots predominantly distributed over the lungs [30], and radioactivity over the thorax was measured continually. Saline alone (0.5 ml per animal), saline containing SMTP-7 (5 mg kg^-1^), scu-PA (250 U kg^-1^), or both SMTP-7 and scu-PA was intravenously injected ~20 min after the embolization. The monitoring of radioactivity was continued further for ~20 min. The fractional change in radioactivity during 20 min after the treatments represented the rate of clot dissolution.

### Bleeding and rebleeding in mice

The measurement of bleeding time and rebleeding volume (secondary oozing from the bleeding time wounds) were performed using male ICR mice (6 weeks of age) as described previously [31]. Briefly, mice were anesthetized with 60 mg kg^-1 ^pentobarbital intraperitoneally and given bolus injection of 5% mannitol (5 ml kg^-1^) or SMTP-7 (5 and 30 mg kg^-1^) in 5% mannitol *via *a tail vein. Five minutes after the administration, a 5-mm tail segment was amputated with a razor blade. The tail was immersed immediately in prewarmed saline at 37°C, and the time required to stop visual spontaneous bleeding was determined. To evaluate rebleeding, the tail was then immersed in another 4-mL prewarmed saline (37°C) containing 14 mM trisodium citrate for 60 min. Red blood cells were collected and lysed in water to measure absorbance at 490 nm, from which blood loss by rebleeding was calculated.

## List of abbreviations

t-PA: tissue-type plasminogen activator; u-PA: urokinase-type plasminogen activator; scu-PA: single-chain urokinase-type plasminogen activator; Pm-AP: plasmin-α_2_-antiplasmin complex.

## Competing interests

The authors declare that they have no competing interests.

## Authors' contributions

WH carried out the animal studies. RN carried out the biochemical studies. NN performed the *ex vivo *studies and the bleeding assays. KH participated in the design and coordination of the study and drafted the manuscript. All authors read and approved the final manuscript.

## Authors' information

HW is currently a Professor, Weifang Medical University, Shangdong, China. RN is currently a post doc fellow at W. M. Keck Center for Transgene Research, University of Notre Dame, IN, USA. NN is a research scientist of TMS Co., Ltd. KH is a Professor, Department of Applied Biological Science, Tokyo Noko University, and President of TMS Co., Ltd.

## Supplementary Material

Additional file 1 **Supplementary Methods and Supplementary Table 1**. Click here for file

Additional file 2 **Supplementary Figure 1: The bases of the quantification of Pm-AP and the confirmation of its authenticity**. (A) Human Pm-AP (*hPm-AP*) was resolved on a SDS-polyacrylamide gel containing 2 mg ml^-1 ^human fibrinogen and processed for detection of lysis band. The plotted data are the average of duplicate determinations. The intensity of the lysis bands at ~140 kDa was proportional to the amount of hPm-AP at 0.1-3 ng (*r *= 0.99). (B) Mouse citrated plasma was recalcified (20 mM CaCl_2_) for 16 h at room temperature to induce Pm-AP formation. After centrifugation, the resulting supernatant (5 μl) was mixed for 30 min with 0.6 μg ml^-1 ^of anti-plasminogen IgG (*Anti-Plg*) or anti-α_2_-antiplasmin IgG (*Anti-AP*). After incubation for 30 min at room temperature, supernatant of the sample (equivalent to 2 μl of the original plasma) was resolved on a SDS-polyacrylamide gel containing 2 mg ml^-1 ^casein for zymography. The intensity of the lysis band at ~130 kDa was decreased by the treatments with anti-plasminogen IgG and anti-α_2_-antiplasmin IgG, demonstrating the authenticity of the ~130 kDa lysis band as Pm-AP. Similar results were obtained when samples were processed for zymography after removal of the immune complexes with protein G-Sepharose. (It is likely that the immune complexes precipitate following the reaction or that the IgG binding prevents the regeneration of plasmin activity during the zymography process.) Click here for file

Additional file 3 **Supplementary Figure 2: Bleeding and rebleeding following SMTP-7 administration in mice**. (A) Bleeding time in mice was determined after an intravenous administration of 5% mannitol (*Control*) or SMTP-7 (5 and 30 mg kg^-1^) dissolved in the vehicle. (B) Following the determination of bleeding time, the mice were subjected to the measurement of rebleeding. The mean + SD (*n *= 7 for control and 5 mg kg^-1 ^groups and *n *= 5 for 30 mg kg^-1 ^group) was shown. There was no statistical difference between any groups with respect to both bleeding time and rebleeding volume as assessed by the Tukey-Kramer test. Click here for file
