# Supplementary material for: SMTP (Stachybotrys microspora triprenyl phenol) enhances clot clearance in a pulmonary embolism model in rats
Source: Thromb J. 2012 Jan 9;10:2. doi: 10.1186/1477-9560-10-2 (PMC3310738; doi:10.1186/1477-9560-10-2)
Supplement: Additional file 1 — Supplementary Methods and Supplementary Table 1. [file 1477-9560-10-2-S1.PDF]

Additional file 1

Supplementary Methods

Production of SMTP congeners

S. microspora IFO 30018 was aerobically incubated at 25 °C in the production medium as described previously [19]. After incubation for 96 h, a precursor amine (L-cystine for SMTP-9, 1,4-butanediamine for SMTP-30, or D,L-2,6-diaminopimelic acid for SMTP-31) was added to the culture at 1 mg ml^{-1}, and incubation was continued for 40 h.

Isolation of SMTP congeners

The culture was mixed with 2 volumes of methanol, and the mixture was filtered and concentrated. SMTP congeners were precipitated at pH 2 and dissolved in methanol. The sample was subjected to preparative HPLC with a solvent system of methanol in 50 mM ammonium acetate (the methanol concentrations (vol/vol) were 70% for SMTP-9, 85% for SMTP-30, and 75% for SMTP-31). Fractions containing each congener were evaporated and extracted with ethyl acetate. The yields of SMTP-9, -30, and -31 were 39, 345, and 59 mg per liter, respectively. The structures of these congeners were confirmed by spectroscopic analyses as shown in Fig. 1A (*see* Supplementary Table 1 for the summary of the spectral data).

Spectroscopic methods

UV spectrum was measured in MeOH on a model 320 spectrometer (Hitachi, Tokyo, Japan) and IR spectrum on a JIR-WINSPEC (JEOL, Tokyo, Japan) with NaCl. Matrix-assisted laser desorption/ionization time-of-flight mass spectrum was taken on a Voyager DE STR (Applied Biosystem, CA, USA) using α-cyano-4-hydroxycinnamic acid as a matrix.

Supplementary Table 1

Supplementary Table 1. Physicochemical properties of SMTP-9, -30, and -31

	SMTP-9	SMTP-30	SMTP-31
Appearance	Light brown oil	Light brown oil	Pale yellow oil
Molecular formula	$C_{52}H_{68}N_2O_{12}S_2$	$C_{50}H_{68}N_2O_8$	$C_{53}H_{70}N_2O_{12}$
MALDI-TOF-MS			
Found $(M + H)^+$	977.33	825.52	927.50
Calculated	977.43 for $C_{52}H_{69}N_2O_{12}S_2$	825.51 for $C_{50}H_{69}N_2O_8$	927.50 for $C_{53}H_{71}N_2O_{12}$
UV λ_{max} nm (ε) MeOH	215 (84,170)<br>260 (20,020)<br>300 (6,100)	214 (88,716)<br>258 (18,469)<br>300 (6,761)	215 (138,603)<br>259 (32,057)<br>300 (9,821)
IR ν_{max} (neat) cm^{-1}	3420, 2920, 2950, 1680, 1570, 1410, 1060	3280, 2970, 2922, 2862, 1664, 1616, 1464, 1342, 1248, 1211, 1157, 1074, 849, 771	3292, 2968, 2922, 2862, 1713, 1666, 1618, 1464, 1356, 1211, 1167, 1074, 849, 775
