# Supplementary material for: SMTP (Stachybotrys microspora triprenyl phenol) enhances clot clearance in a pulmonary embolism model in rats
Source: Thromb J. 2012 Jan 9;10:2. doi: 10.1186/1477-9560-10-2 (PMC3310738; doi:10.1186/1477-9560-10-2)
Supplement: Additional file 2 — Supplementary Figure 1: The bases of the quantification of Pm-AP and the confirmation of its authenticity. (A) Human Pm-AP (hPm-AP) was resolved on a SDS-polyacrylamide gel containing 2 mg ml-1 human fibrinogen and processed for detection of lysis band. The plotted data are the average of duplicate determinations. The intensity of the lysis bands at ~140 kDa was proportional to the amount of hPm-AP at 0.1-3 ng (r = 0.99). (B) Mouse citrated plasma was recalcified (20 mM CaCl2) for 16 h at room temperature to induce Pm-AP formation. After centrifugation, the resulting supernatant (5 μl) was mixed for 30 min with 0.6 μg ml-1 of anti-plasminogen IgG (Anti-Plg) or anti-α2-antiplasmin IgG (Anti-AP). After incubation for 30 min at room temperature, supernatant of the sample (equivalent to 2 μl of the original plasma) was resolved on a SDS-polyacrylamide gel containing 2 mg ml-1 casein for zymography. The intensity of the lysis band at ~130 kDa was decreased by the treatments with anti-plasminogen IgG and anti-α2-antiplasmin IgG, demonstrating the authenticity of the ~130 kDa lysis band as Pm-AP. Similar results were obtained when samples were processed for zymography after removal of the immune complexes with protein G-Sepharose. (It is likely that the immune complexes precipitate following the reaction or that the IgG binding prevents the regeneration of plasmin activity during the zymography process.) [file 1477-9560-10-2-S2.PDF]

Additional file 2

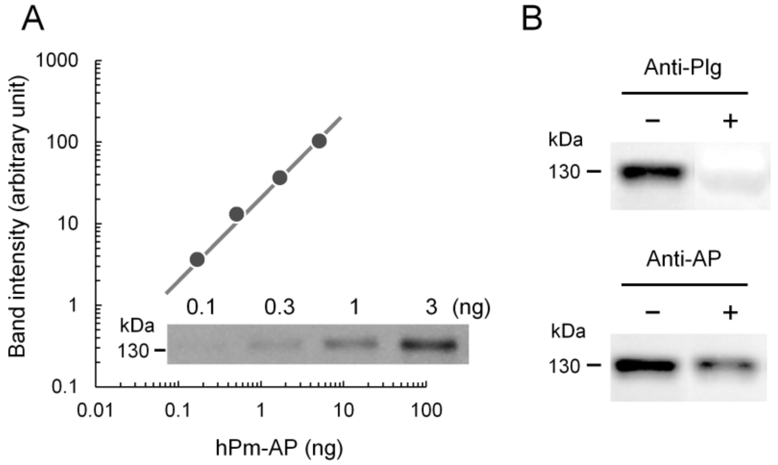

Supplementary Fig. 1. The bases of the quantification of Pm-AP and the confirmation of its authenticity.

(A) Human Pm-AP (*hPm-AP*) was resolved on a SDS-polyacrylamide gel containing 2 mg ml^{-1} human fibrinogen and processed for detection of lysis band. The plotted data are the average of duplicate determinations. The intensity of the lysis bands at ~140 kDa was proportional to the amount of hPm-AP at 0.1–3 ng (r = 0.99). (B) Mouse citrated plasma was recalcified (20 mM $CaCl_2$) for 16 h at room temperature to induce Pm-AP formation. After centrifugation, the resulting supernatant (5 μl) was mixed for 30 min with 0.6 mg ml^{-1} of anti-plasminogen IgG (*Anti-Plg*) or anti-α_2-antiplasmin IgG (*Anti-AP*). After incubation for 30 min at room temperature, supernatant of the sample (equivalent to 2 μl of the original plasma) was resolved on a SDS-polyacrylamide gel containing 2 mg ml^{-1} casein for zymography. The intensity of the lysis band at ~130 kDa was decreased by the treatments with anti-plasminogen IgG and anti-α_2-antiplasmin IgG, demonstrating the authenticity of the ~130 kDa lysis band as Pm-AP. Similar results were obtained when samples were processed for zymography after removal of the immune complexes with protein G-Sepharose. (It is likely that the immune complexes precipitate following the reaction or that the IgG binding prevents the regeneration of plasmin activity during the zymography process.)
