# Supplementary material for: SMTP (Stachybotrys microspora triprenyl phenol) enhances clot clearance in a pulmonary embolism model in rats
Source: Thromb J. 2012 Jan 9;10:2. doi: 10.1186/1477-9560-10-2 (PMC3310738; doi:10.1186/1477-9560-10-2)
Supplement: Additional file 3 — Supplementary Figure 2: Bleeding and rebleeding following SMTP-7 administration in mice. (A) Bleeding time in mice was determined after an intravenous administration of 5% mannitol (Control) or SMTP-7 (5 and 30 mg kg-1) dissolved in the vehicle. (B) Following the determination of bleeding time, the mice were subjected to the measurement of rebleeding. The mean + SD (n = 7 for control and 5 mg kg-1 groups and n = 5 for 30 mg kg-1 group) was shown. There was no statistical difference between any groups with respect to both bleeding time and rebleeding volume as assessed by the Tukey-Kramer test. [file 1477-9560-10-2-S3.PDF]

Additional file 3

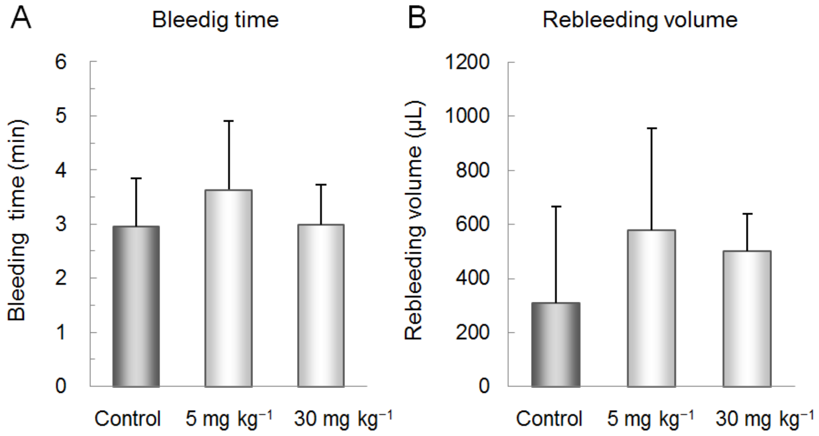

Supplementary Fig. 2. Bleeding and rebleeding following SMTP-7 administration in mice.

(A) Bleeding time in mice was determined after an intravenous administration of 5% mannitol (*Control*) or SMTP-7 (5 and 30 mg kg^{-1}) dissolved in the vehicle. (B) Following the determination of bleeding time, the mice were subjected to the measurement of rebleeding. The mean + SD (n = 7 for control and 5 mg kg^{-1} groups and n = 5 for 30 mg kg^{-1} group) was shown. There was no statistical difference between any groups with respect to both bleeding time and rebleeding volume as assessed by the Tukey-Kramer test.
